# Mammogram mastery: Breast cancer image classification using an ensemble of deep learning with explainable artificial intelligence

**DOI:** 10.1097/MD.0000000000042242

**Published:** 2025-05-30

**Authors:** Proloy Kumar Mondal, Md. Khurshid Jahan, Haewon Byeon

**Affiliations:** a Discipline of Electronics and Communication Engineering, University of Khulna, Khulna, Bangladesh; b Department of Electrical and Computer Engineering, North South University, Dhaka, Bangladesh; c Worker’s Care and Digital Health Lab, Department of Future Technology, Korea University of Technology and Education, Cheonan, South Korea.

**Keywords:** breast cancer, deep learning, ensemble models, explainable AI, image classification, mammogram mastery

## Abstract

Breast cancer is a serious public health problem and is one of the leading causes of cancer-related deaths in women worldwide. Early detection of the disease can significantly increase the chances of survival. However, manual analysis of mammogram mastery images is complex and time-consuming, which can lead to disagreements among experts. For this reason, automated diagnostic systems can play a significant role in increasing the accuracy and efficiency of diagnosis. In this study, we present an effective deep learning (DL) method, which classifies mammogram mastery images into cancer and noncancer categories using a collected dataset. Our model is pretrained based on the Inception V3 architecture. First, we run 5-fold cross-validation tests on the fully trained and fine-tuned Inception V3 model. Next, we apply a combined method based on likelihood and mean, where the fine-tuned Inception V3 model demonstrated superior performance in classification. Our DL model achieved 99% accuracy and 99% F1 score. In addition, interpretable AI techniques were used to enhance the transparency of the classification process. The finely tuned Inception V3 model demonstrated the highest performance in classification, confirming its effectiveness in automatic breast cancer detection. The experimental results clearly indicate that our proposed DL-based method for breast cancer image classification is highly effective, especially its application in image-based diagnostic methods. This study brings to the fore the huge potential of AI-based solutions, which can play a significant role in increasing the accuracy and reliability of breast cancer diagnosis.

## 1. Introduction

Cancer is considered a serious global public health problem. Among various cancers, the rate of breast cancer in women ranks second highest after lung cancer. Also, the mortality rate of breast cancer is quite high compared to many types of cancer.^[1]^ Despite rapid advances in medical science, histopathological image analysis is still the most widely used method for breast cancer diagnosis.^[2]^ The classification task is of particular importance in this analysis, as accurate and automated classification of high-resolution histopathological images forms the basis of studies such as nucleus localization, mitosis detection and gland segmentation.

Currently, histopathological imaging in clinical practice is largely dependent on manual qualitative analysis by pathologists. However, this approach involves 3 major problems. First, there is a shortage of pathologists globally, especially in underdeveloped regions and in smaller hospitals. Scarcity and uneven distribution of these resources remains an immediate problem. Second, the accuracy of histopathological diagnosis is completely dependent on the skill and long-term experience of the pathologist, which often results in diagnostic inconsistency. Third, the complexity of histopathological imaging makes pathologists tired and inattentive. To solve these problems, accurate and automated histopathological image analysis methods, especially classification techniques, need to be developed.

**Figure 1.**
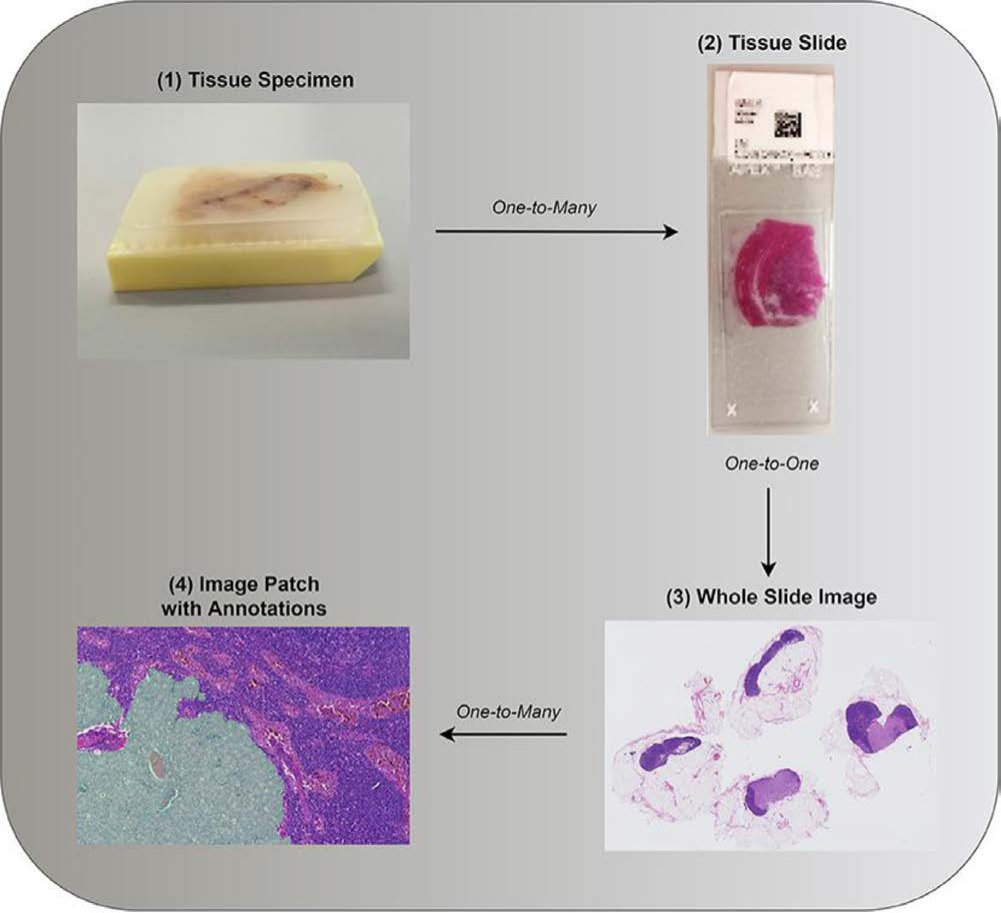
The complete process of biopsy.^[7]^ Pathologists examine these slides under a microscope to diagnose breast cancer.^[7]^ The biopsy procedure is shown in the figure and described in detail in the study by Dimitriou et al.^[7]^

Cancer is considered a serious public health problem worldwide. According to the Global Burden (GBD) 2017 survey, 24.5 million cancer cases and 9.6 million cancer-related deaths were recorded worldwide that year. These data indicate a 33% increase in cancer incidence between 2007 and 2017.^[3]^ In particular, breast cancer is the most common malignancy in women and the leading cause of cancer-related death.^[3,4]^ Therefore, early detection of breast cancer plays an important role in preventing the progression of the disease and reducing its incidence among women. Breast cancer is a multidimensional disease, consisting of different biological, histological and clinical features.^[5]^ The growth of this malignancy is capable of invading abnormal breast cells and adjacent normal tissue. Its clinical screening is usually conducted by radiological imaging, such as mammography, ultrasound and magnetic resonance imaging.^[6]^ However, these noninvasive imaging methods may not always be able to accurately identify cancerous areas. To address this challenge, biopsy procedures are commonly used to analyze the malignancy of breast cancer tissue in greater depth. In the biopsy procedure, tissue samples are collected and placed on microscopic slides and the slides are stained to clearly show the nucleus and cytoplasm.^[2]^ Then, pathologists examine these slides under a microscope to diagnose breast cancer.^[7]^ The biopsy procedure is shown in Figure 1 and described in detail in the study by Dimitriou et al.^[7]^

However, manual analysis of complex histopathological images is time-consuming and tedious, which carries the risk of errors. In addition, the morphological criteria used in the classification of these images are somewhat subjective, resulting in an average diagnostic agreement between pathologists of approximately 75% of cases.^[8]^ For this reason, computer-aided diagnosis^[9]^ plays an important role in assisting pathologists in the analysis of histopathological images, especially as it increases the accuracy of breast cancer diagnosis and reduces diagnostic variation among pathologists.^[2]^ Yet, conventional computerized diagnostic methods ranging from rule-based systems to machine learning techniques fail to effectively address the challenges of intraclass variation and consistency in histopathology.^[9]^ Also, these methods rely on techniques such as scale-invariant feature transform,^[10]^ speed barest feature,^[11]^ and local binary pattern^[12]^ for feature extraction, all of which are based on supervised information and result in biased results during classification. There are risks.^[13]^ This, in turn, leads to the need for advanced computational models based on multiple layers of nonlinear processing units for an efficient diagnosis, called deep learning (DL).^[14]^

Recently, DL models have made significant progress in computer vision, especially in biomedical image processing, which has motivated various researchers to use them in the classification of breast cancer histopathology for their ability to automatically learn complex features and extract advanced features from images.^[7]^ In particular, convolutional neural network (CNN)^[15]^ is widely used in image-related tasks, because of its ability to share parameters at different layers. Numerous CNN-based architectures have been proposed in recent years, but AlexNet^[16]^ is considered the first deep CNN, which achieved significant accuracy in the 2012 imagenet large scale visual recognition challenge (ILSVRC). Later, the VGG architecture^[17]^ introduced the concept of deeper networks with small convolutional filters and won second place in ILSVRC 2014. Stacking of small convolutional filters can provide an efficient receptive field these insights have been used in recently proposed models such as inception networks^[18]^ and residual neural networks.^[19]^ In this study, we presented efficient classification of breast cancer dataset using DL methods of Inception V3 architecture.

Next, we selected and trained of inception V3^[20]^ DL architectures (discussed in Section 3). Specifically, we evaluated both the individual and ensemble performances of the fully-trained and fine-tuned Inception V3 frameworks.^[20]^ Our main goal was to ensure accurate classification of the class, and we observed that the combination of fine-tuned Inception V3 methods^[20]^ provided improved performance in the classification of cancer and noncancer images.

The remainder of this paper is organized as follows: Section 2 presents related work. Section 3 describes the materials and methods used. Section 4 discusses the experimental setup and Section 5 provides results and discussion. Finally, Section 6 provides the conclusion of this study and future directions.

## 2. Related works

With the advancement of ML in biomedical engineering, many studies have been conducted on hand-crafted feature-based methods for classification of breast histopathology images. For example, Kowal et al^[21]^ extracted 42 features related to shape, topology, and texture from segmented nuclei of 500 fine-needle biopsies with emphasis on nucleus segmentation, which were used to train 3 classifiers for breast cancer image classification. Similarly, Filipczuk et al^[22]^ studied segmented nuclei and determined 25 shape and texture-based features from 737 cytology images and trained 4 ML classifiers such as K-Nearest Neighbor (KNN), Naive Bayes, Decision Tree, and support vector machines.

Some studies have also focused on extracting global features from the whole image. For example, Zhang et al^[23]^ designed a cascade random space ensemble by combining different features that can effectively classify microscopic biopsy images of breast cancer. Although traditional ML methods have shown satisfactory performance in histological image analysis of breast cancer, their performance is dependent on feature selection. Also, traditional methods cannot always effectively extract and organize features. On the other hand, DL models have the ability to extract complex and high-level features, which are automatically extracted from images. Therefore, most of the recent studies have used DL methods, especially with and without pretrained models. Several studies have demonstrated the effectiveness of this approach using the Breakhis dataset.^[24]^ For example, Spainhol et al^[25]^ improved the accuracy of Breckhis dataset using CNN in breast cancer histopathology image classification. In this study, we Araújo et al^[26]^ and Yan et al,^[27]^ We used a DL model following the approach of the study by Yan et al.^[27]^ Vang et al^[28]^ first proposed the use of Google’s Inception-V3 model to perform patch-based classification. These patch-based predictions were then passed through an ensemble fusion structure involving majority voting, a gradient boosting machine, and logistic regression to obtain an image-based prediction. On the other hand, Rakhalin et al^[29]^ suggested a different approach named Deep Rotated Feature Representation. In this method, pathological images were initially encoded using a standard CNN to obtain a low-dimensional sparse representation (either 1408 or 2048 dimensions). However, our dataset contains only cancer and noncancer classes.

## 3. Materials and methods

In this section, we present our dataset, discuss its preprocessing methods, and test criteria including training, validation, and augmentation processes. Next, we describe the layout of the Inception V3 model and the implementation of interpretable AI, and finally, detail the structure of our proposed ensemble architecture.

### 3.1. Dataset collection

This dataset represents an extensive collection of breast cancer images, divided into 2 distinct groups: one of patients with cancer and the other of healthy individuals.^[30]^ To ensure reliability and accuracy for research and education, the dataset has been carefully curated, verified and classified by expert clinicians. Based on data collected from the Sulaymaniyah region of Iraq, this dataset provides a unique perspective on breast cancer characteristics and prevalence in the region. The dataset contains 745 original images and 9685 enhanced images, which serve as an important resource for training and evaluating DL algorithms for breast cancer detection. The extensive collection of X-ray images provides additional versatility for algorithm development and educational efforts. This dataset has significant potential to advance medical research, develop innovative diagnostic tools, and expand educational opportunities for medical students interested in breast cancer diagnosis. The dataset consists of images classified into 2 categories, noncancer and cancer in Table 1. It contains 1487 noncancer images and 290 cancer images, for a total of 1777 images. All images use the RGB color model and no staining method is applied, as it is not applicable to this dataset.

**Table 1:** Characteristics of our proposed dataset.

Image	Quantity	Color model	Staining
Non-cancer	1487	RGB	N/A
Cancer	290	RGB	N/A
Total	1777	RGB	N/A

### 3.2. Preprocessing

To prepare the dataset for training, we first performed a preprocessing step, which included resizing all images to the dimensions required by the Inception V3 model and normalizing the pixel values to match the model’s expected input range. It maintains consistency in data format and optimizes it for efficient processing.

**Figure 2.**
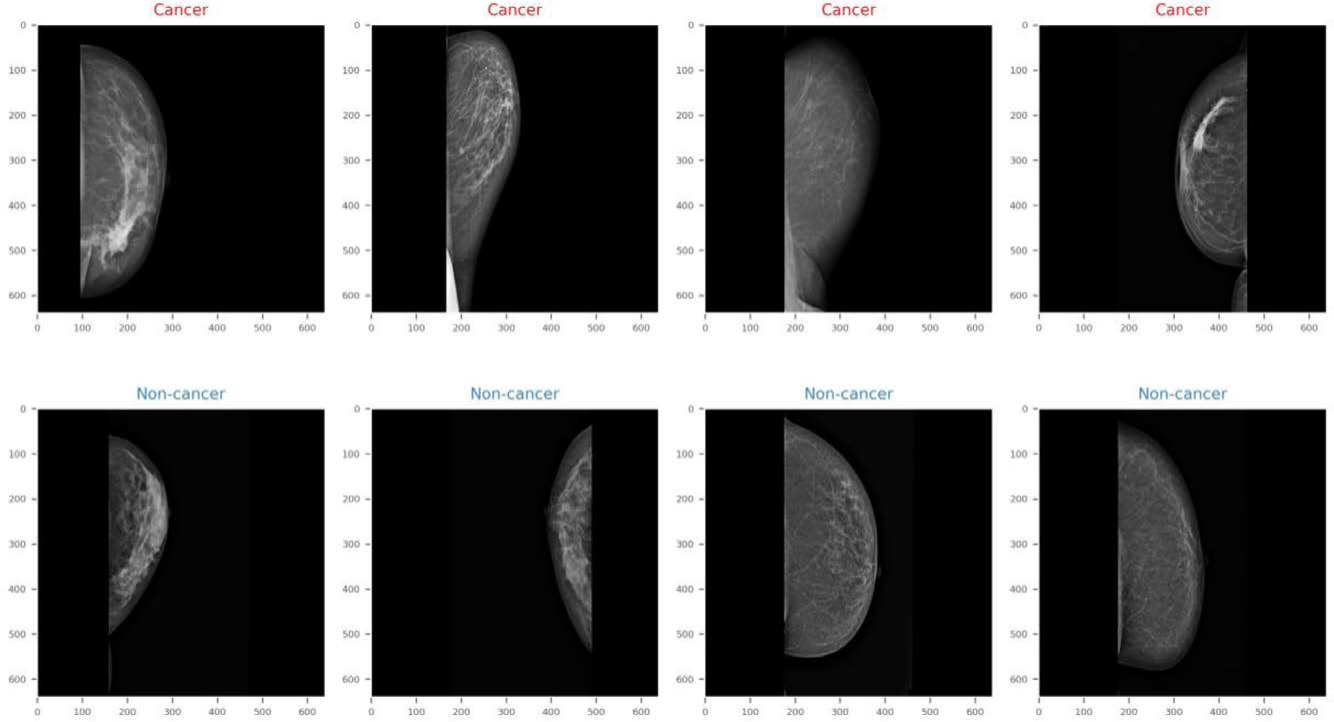
Samples from the original dataset. The graphic depicts mammography scans divided into cancer (top row, red label) and noncancer (bottom row, blue label) groups. The carcinogenic scans most likely show abnormal growths or density alterations, while the noncancerous images show normal breast tissue without malignancy.

The dataset used in this study includes stained images of breast cancer, which are widely used to aid pathologists during evaluation. However, it is difficult to maintain the same spot density in all images, resulting in color variations in the images. This color variation can have a negative impact on the training of CNN models, so color normalization is usually applied. In this study, we used the method proposed by^[31]^ for color normalization following a recent study.^[26]^ In this method, images are first converted to optical density (OD) by logarithmic transformation. Next, 2-dimensional high variance estimates are extracted from the OD tuples by applying singular value decomposition. Later, this color space transformation is applied to the original images. Finally, the images are enhanced by 90% data enhancement. However, the use of normalized images reduced the classification performance of our proposed model, as explained in detail [34]. Therefore, we used the original images without stain normalization in this study. Examples of noncancerous and cancerous images are shown in Figure 2.

### 3.3. Traning criteria

For the individual and combined models, we kept 80% of the total images for training and the remaining 20% for testing, where the proportion of cancer and noncancer images was the same. Thus, 1703 images were selected for training and 74 resampled images for testing. According to the guidelines of the study by Yao et al,^[32]^ we applied 5-fold cross-validation on the training images, of which 1557 images were used for training and 146 images were used for validation. Here, the proportion of cancer and noncancer images is kept the same in both training and validation. These statistics for training, validation, and testing are shown in Table 2.

**Table 2:** Criteria for selecting training, validation, and test images.

	No. of images	Percentage
Training	1557	87.6
Validation	146	8.2
Testing	74	4.2
Total	1777	100

### 3.4. Data augmentation

Image data augmentation is a technique used to augment datasets by creating modified images in the training process. Using Keras DL library’s ImageDataGenerator,^[33]^ we generated batches of tensor image data through real-time data augmentation. With this augmentation method, our goal was to allow the network to see a new variety of data every epoch. First, an input batch is provided to the ImageDataGenerator, which applies transformations like random translation, rotation, etc to each image. We set “rotation_range = 40,” which is for random rotation between (−40, 40) degrees, and used “width_shift_range = 0.2” and “height_shift_range = 0.2,” which define random shifts of the image by fractions of width and height. If the rotation causes the pixel to go out of frame, we use the “reflect” mode to fill the gap. The randomized batch is then returned to the calling function, and its parameters and values are shown in Table 3.

**Table 3:** Parameters of data augmentation.

Parameters of image augmentation	Values
Zoom range	0.2
Width shift range	0.2
Height shift range	0.2
Horizontal flip	True

### 3.5. Inception V3 architecture

A CNN is a method specialized in image data classification,^[34]^ especially for image recognition problems.^[35]^ A key advantage of the CNN model is the layered learning structure that enables matching the model topology to the input features, and improves model accuracy by reducing the number of parameters by analyzing local visual patterns.^[36]^ Inception V3 is a CNN architecture developed by Google for image classification tasks and is the third version of the Inception architecture, which was developed in 2015 by Szegedy et al was introduced by.^[37]^ Inception V3 is designed to improve performance and image classification performance based on previous architectures. It consists of multiple interconnected inception modules, which extract features from the input image by combining various convolution and pooling layers.

**Figure 3.**
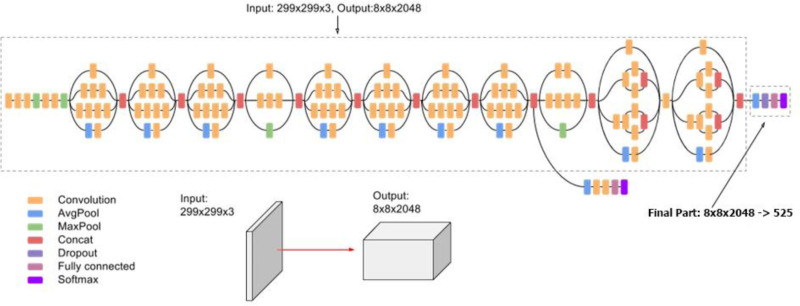
Architecture of Inception-V3. This is an example of a deep convolutional neural network, where an input image of size 299 × 299 × 3 flows through multiple layers – convolution (orange), pooling (blue and green), concatenation (red), dropout (purple), and a fully connected layer (magenta). Step by step, the network learns features by reducing spatial dimension and increasing depth, eventually producing an output tensor of size 8 × 8 × 2048. In the final stage, this feature representation is transformed into 525 output classes using fully connected and softmax layers.

The Inception V3 model consists of 484 layers and contains 11 inception modules, with an input size of 299 × 299 pixels. Each inception module consists of convolution filter, pooling layer and ReLu activation function. InceptionV3 reduces the number of parameters using factorized convolution without reducing network efficiency. Besides, InceptionV3 also offers a new dimension reduction technique to reduce the number of features. Figure 3 depicts our fine-tuned inceptionV3 model for classification of Breast cancer. Another important feature is batch normalization, which helps to normalize input data and reduce internal variation during training. The Inception V3 model is pretrained on the ImageNet dataset, which allows for transfer learning and can be effectively applied to small datasets of various classes. This is helpful in saving a lot of data and calculations instead of building new models from scratch.

Inception V3 has shown good results when used in a variety of applications including image classification, object detection, and image segmentation. Its factorized convolution and inception modules make this model highly efficient and accurate. Also, it can be used versatile in various applications due to being pretrained on ImageNet dataset, which is also quite improved in accuracy. The Inception V3 architecture, while more complex, is smaller and faster to model than VGG with about 4 million parameters, which uses a pooling layer instead of a fully coupled layer. Figure 3 represents the workflow of Inception V3 architecture.

## 4. Experimental setup

In this section, we provide a detailed description of the test environment and then explain the evaluation metrics used in our proposed model. Next, we outline the hyperparameter tuning process and finally implement interpretable AI techniques.

### 4.1. Implementation

We implemented all the experiments in this study on a standard PC, with dual Nvidia GeForce RTX 4080 graphical processing unit (GPU) support was selected for this study for its high computational power, which speeds up the training and evaluation of DL models. The RTX 4080 features 16,384 CUDA cores and 16 GB of GDDR6X memory, capable of efficiently handling large mammogram image datasets and complex neural network architectures. Compared to alternative GPUs such as the RTX 3080 or RTX 3090, the RTX 4080 offers improved performance per watt, faster processing speed, and better support for DL-intensive tasks. These features help reduce training time and improve model performance, making it the best choice for this study.

Python 3.12.0, TensorFlow 2.14.1, and Keras 2.2.4 installed. Also, this PC has a Ryzen 7 5700x processor with 32.0 GB RAM and 16 logical threads and 32MB cache memory.

### 4.2. Evaluation metrics

The overall performance of our proposed model depends on the elements of confusion matrix, also known as error matrix or confusion matrix. This evaluation matrix has 4 components: true positive (TP), false positive (FP), false negative (FN), and true negative (TN). In our case, TP refers to images correctly classified as cancerous, FP to noncancerous images incorrectly classified as cancerous, FN to cancerous images correctly classified as noncancerous, and TN to noncancerous images correctly classified. Based on the confusion matrix, we used 4 performance indicators to evaluate the model’s classification performance: precision, sensitivity (recall), overall accuracy, and F1-score, which are calculated using Python Skit-Learn module. The formula required for this performance measurement is given below, and the evaluation metrics are determined using equations (1) to (4).

**Precision:** It measures the accuracy of the model and represents the proportion of images correctly classified among the predicted carcinoma images.

$$\begin{array}{l} Precision=\frac{TP}{TP+FP} \end{array}$$ (1)

**Sensitivity:** Also known as “recall,” this measures the completeness of the model. It indicates the proportion of images correctly classified as carcinoma out of total carcinoma images.

$$\begin{array}{l} Sensitivity=\frac{TP}{TP+FN} \end{array}$$ (2)

**Overall accuracy:** It evaluates the accuracy of the model and indicates the proportion of images correctly classified out of the total images tested.

$$\begin{array}{l} Accuracy=\frac{TP+TN}{TP+FP+TN+FN} \end{array}$$ (3)

**F1-score:** It represents the harmonic mean of precision and recall and is commonly used to balance precision and recall in model optimization.

$$\begin{array}{l} F1\text{-}Score=2\times \frac{precision\times recall}{precision+recall} \end{array}$$ (4)

### 4.3. Hyperparameter tuning

The hyperparameter tuning for the Inception V3 model with data augmentation involves several key settings. The training follows a standard approach, using the Adam optimizer, which includes weight decay to help prevent overfitting by discouraging large weights. The loss function is cross-entropy, which is suitable for classification tasks. A learning rate of 0.0005 is set, allowing the model to learn gradually without drastic updates. The batch size is 16, meaning the model processes 16 images at a time, balancing memory usage and convergence speed. Convolution layers include 1 × 1, 3 × 3, and 5 × 5 filters within the Inception modules, with padding set to “Same” to maintain the output size. Global average pooling is applied for pooling, helping reduce feature dimensions before the fully connected layers. The model is trained over 10 epochs, and the architecture is fine-tuned to adapt pretrained layers to the specific dataset used here. For a clearer understanding, this information is presented in Table 4, which outlines the hyperparameter settings in detail.

**Table 4:** Hyperparameters used in the individual and an ensemble model.

Hyperparameters	Inception V3 with data augmentation
Train approach	Standard
Optimizer	Adam
Loss function	Cross-entropy
Learning rate	0.0005
Batch size	16
Convolution	1 × 1, 3 × 3, 5 × 5
Padding	Same
Pooling	Global average pooling
Epochs	10
Architecture	Fine-tuned

### 4.4. Explainable AI

We used interpretable AI (XAI) techniques to increase the clarity and interpretability of our Inception V3 model. Interpretable AI is important in understanding the decision-making process of DL models, especially in sensitive fields like medical imaging. The interpretable AI model works by identifying which parts of the mammogram image to focus on, especially when it makes predictions. This is accomplished by methods such as Grad-CAM (gradient-weighted class activation mapping) or SHAP (shapley additive explanation), which visualize the image parts that contribute most to the model’s output. By overlaying heatmaps, the XAI method highlights areas that the model identifies as signs of cancer, allowing clinicians to better understand and trust the model’s conclusions.

In this study, interpretable AI not only helps verify the accuracy of predictions, but also ensures that the model is focusing on clinically important features. This interpretability helps keep model conclusions consistent with clinical knowledge, which is crucial for a reliable breast cancer detection system.

## 5. Results and discussion

**Figure 4.**
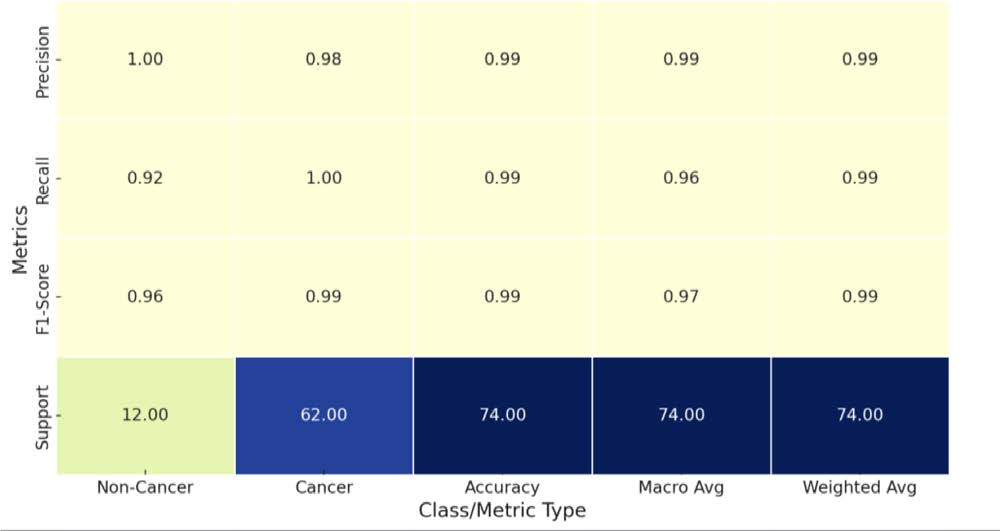
Comprehensive classification metrics results. This illustrates the classification metrics for the inception V3 model, highlighting its performance across precision, recall, and F1-score for both noncancer and Cancer classes, along with the respective support. For the noncancer class, the model achieved a precision of 1.00, indicating no false positives, and a recall of 0.92, reflecting a small number of misclassified instances. The F1-score for noncancer is 0.96, showing strong overall accuracy. In the cancer class, the model demonstrated a precision of 0.98, signifying few FP, and a recall of 1.00, indicating perfect detection of all cancer cases. The F1-score for cancer is 0.99, which confirms the model’s high reliability in predicting cancer cases. The overall accuracy across classes is 0.99, with balanced macro and weighted averages, underscoring the model’s robust performance on the dataset. FP = false positive.

In this part, we evaluated the performance of our proposed DL models based on average predicted probabilities. First, we present the performance metrics of the individual models, and then, we discuss the competitiveness of our proposed models in breast cancer image classification compared with recently published studies. We used the Inception V3 architecture to analyze how the model performs. All images in the processed dataset were used for training. The accuracy of the model was 99%. Figure 4 illustrates the classification metrics for the Inception V3 model, highlighting its performance across precision, recall, and F1-score for both non-Cancer and Cancer classes, along with the respective support. For the noncancer class, the model achieved a precision of 1.00, indicating no FPs, and a recall of 0.92, reflecting a small number of misclassified instances. The F1-score for noncancer is 0.96, showing strong overall accuracy. In the cancer class, the model demonstrated a precision of 0.98, signifying few FP, and a recall of 1.00, indicating perfect detection of all cancer cases. The F1-score for Cancer is 0.99, which confirms the model’s high reliability in predicting cancer cases. The overall accuracy across classes is 0.99, with balanced macro and weighted averages, underscoring the model’s robust performance on the dataset.

**Figure 5.**
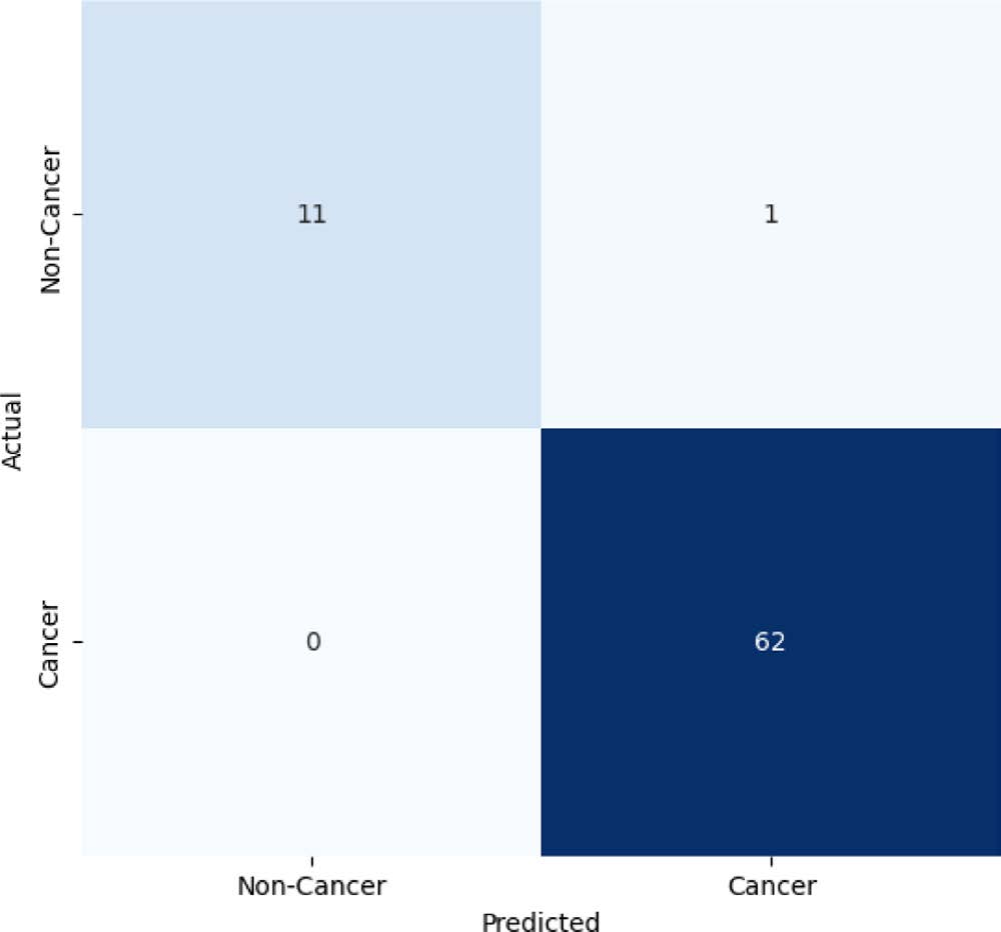
Confusion metrics results outcome. This presents the prediction confusion matrix of the model on the test set, which gives a clear idea of its performance. The model correctly identified 62 examples as “cancer” (TP) and 11 examples as “noncancer” (TN). However, it misclassified a “noncancer” instance as “cancer” (FP) and there were no instances where a “cancer” case was identified as “noncancer” (FN). This reveals the strong accuracy of the model in distinguishing between cancer and noncancer, especially the effective ability of the model in detecting cancer, as reflected by the absence of FNs. FN = false negative, FP = false positive, TN = true negative, TP = true positive.

Figure 5 presents the prediction confusion matrix of the model on the test set, which gives a clear idea of its performance. The model correctly identified 62 examples as “cancer” (TP) and 11 examples as “noncancer” (TN). However, it misclassified a “noncancer” instance as “cancer” (FP) and there were no instances where a “cancer” case was identified as “noncancer” (FN). This reveals the strong accuracy of the model in distinguishing between cancer and noncancer, especially the effective ability of the model in detecting cancer, as reflected by the absence of FNs.

**Figure 6.**
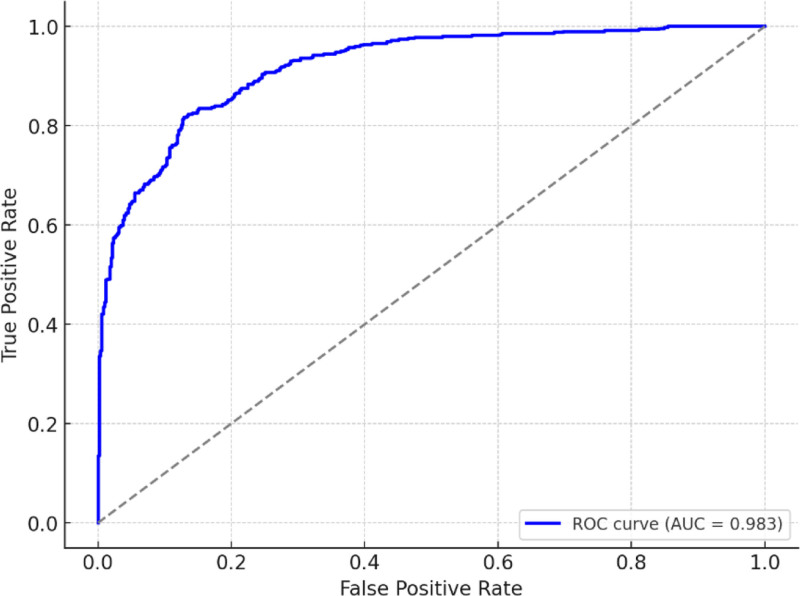
Receiver operating characteristic curve result of classification model. The ROC curve demonstrates the cancer and noncancer classification efficiency of the model. This figure plots the TPR versus the FPR across different threshold values, showing how well the model can distinguish between the 2 classes. In this graph, the orange line represents the ROC curve and it follows the upper bound, indicating perfect classification performance. The AUC is 98.33, which indicates that the model strikes the ideal balance between sensitivity and specificity and correctly identifies all positive cases without false positives. AUC = area under the curve, FPR = false positive rate, ROC = receiver operating characteristic, TPR = true positive rate.

The receiver operating characteristic (ROC) curve in Figure 6 demonstrates the cancer and noncancer classification efficiency of the model. It plots the TP rate versus the FP rate across different threshold values, showing how well the model can distinguish between the 2 classes. In this graph, the orange line represents the ROC curve and it follows the upper bound, indicating perfect classification performance. The AUC is 98.33, which indicates that the model strikes the ideal balance between sensitivity and specificity and correctly identifies all positive cases without FPs.

**Figure 7.**
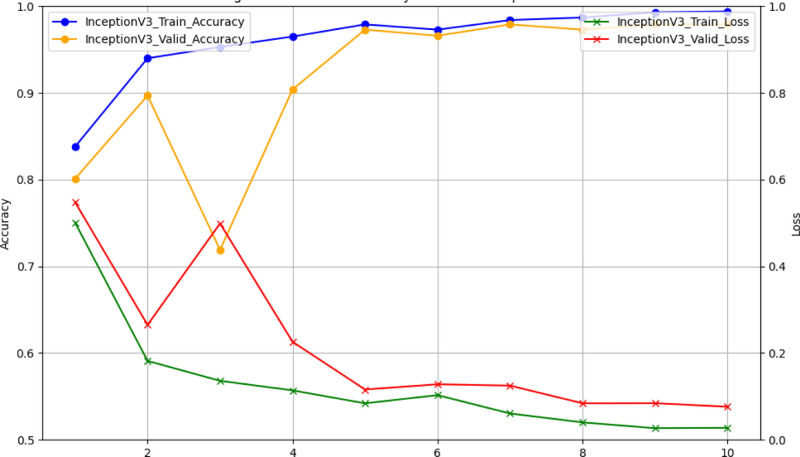
Training and validation accuracy and loss of inception V3 model outcome. This displays the training and validation accuracy and loss over 10 epochs for the Inception V3 model, which gives insight into the model’s learning process and generalization ability. The training accuracy (blue line) increased steadily and reached almost perfect accuracy in the final epoch, indicating that the model learned effectively on the training data. Validation accuracy (yellow line) initially fluctuated but stabilized at a high level, demonstrating good generalizability to unseen data. The training loss (green line) has steadily decreased, showing that the model’s performance on the training set has improved over time. At the same time, the validity loss (red line) decreases initially and stabilizes in the later epochs, consistent with the training loss. This consistency indicates that the model is able to achieve high accuracy on the training and validation sets while avoiding overfitting, maintaining a balance between learning and generalization.

Figure 7 displays the training and validation accuracy and loss over 10 epochs for the Inception V3 model, which gives insight into the model’s learning process and generalization ability. The training accuracy (blue line) increased steadily and reached almost perfect accuracy in the final epoch, indicating that the model learned effectively on the training data. Validation accuracy (yellow line) initially fluctuated but stabilized at a high level, demonstrating good generalizability to unseen data. The training loss (green line) has steadily decreased, showing that the model’s performance on the training set has improved over time. At the same time, the validity loss (red line) decreases initially and stabilizes in the later epochs, consistent with the training loss. This consistency indicates that the model is able to achieve high accuracy on the training and validation sets while avoiding overfitting, maintaining a balance between learning and generalization.

**Figure 8.**
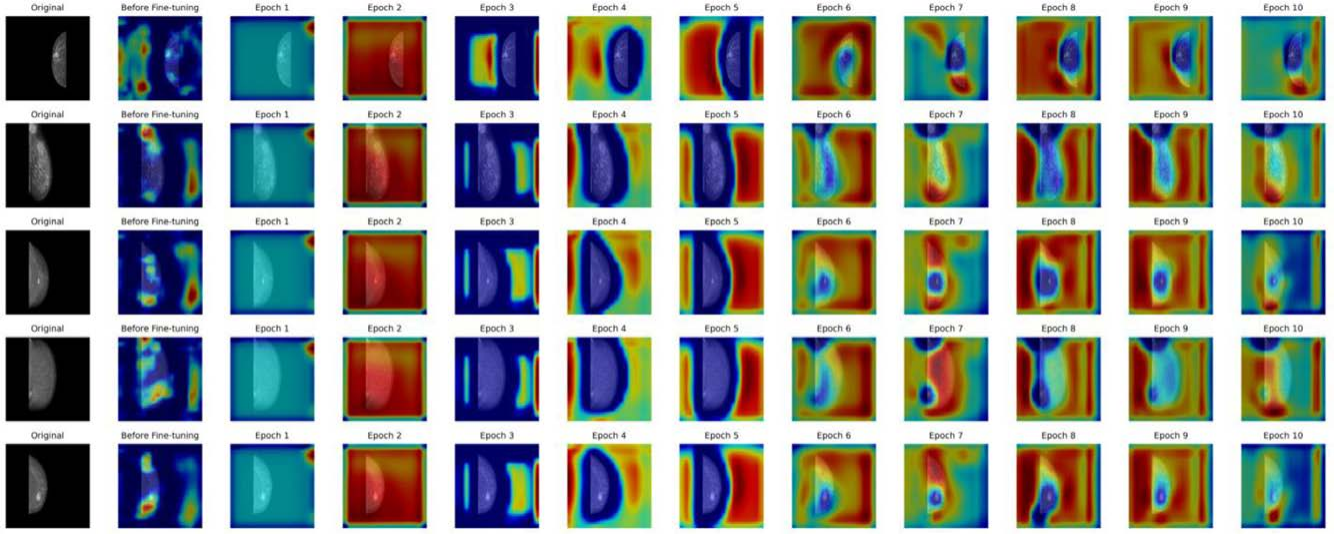
Explainable AI visualization across training epochs for mammogram images. This illustrates the attention fine-tuning of the Inception V3 model over time epochs using interpretable AI (XAI) techniques. Each row corresponds to a mammogram image, showing the continuation of the heatmap at different training stages, starting from the original image. The second column, “before fine-tuning,” displays the model’s initial focus area, which is not clearly aligned with relevant features and is randomly scattered. As training progresses from 1 to 10 epochs, the heatmaps reflect changes in the model’s attention, illustrating the process of progressively focusing on specific areas. These marked regions are likely to be associated with breast cancer characteristics. By the 5th epoch, the model’s attention becomes more stable and begins to focus on relevant areas. In the 10th epoch, the heatmaps show that the model has refined its focus, implying that it has learned to prioritize features important for breast cancer detection.

Figure 8 illustrates the attention fine-tuning of the Inception V3 model over time epochs using interpretable AI (XAI) techniques. Each row corresponds to a mammogram image, showing the continuation of the heatmap at different training stages, starting from the original image. The second column, “before fine-tuning,” displays the model’s initial focus area, which is not clearly aligned with relevant features and is randomly scattered. As training progresses from 1 to 10 epochs, the heatmaps reflect changes in the model’s attention, illustrating the process of progressively focusing on specific areas. These marked regions are likely to be associated with breast cancer characteristics. By the 5th epoch, the model’s attention becomes more stable and begins to focus on relevant areas. In the 10th epoch, the heatmaps show that the model has refined its focus, implying that it has learned to prioritize features important for breast cancer detection.

This XAI approach provides valuable insight into the model’s learning process, showing how it detects and pays attention to relevant patterns. Heatmaps not only increase interpretability but also provide reassurance for clinicians, as they can see which areas of the mammogram influence the model’s predictions at each stage.

## 6. Conclusions

In this paper, we propose an ensemble DL approach for breast classification, which analyzes mammogram mastery images of cancer using our collected dataset. The main goal of this study was to efficiently classify cancer images. We observed that model probabilities can be more efficient with averaging forecasting methods. For this purpose, we used an ensemble of fine-tuned Inception V3 models, which produced relatively robust models. The proposed ensemble method exhibits competitive performance in classifying complex mammogram images. We used XAI to understand the learning process of the model, which demonstrates how attention is focused on identifying relevant patterns. However, our dataset contains only 2-class images. In the future, we plan to expand our dataset and include multi-class images. Also, future studies may consider incorporating other pretrained models. Finally, the application of such an integrated approach to mammogram imaging in other cancers such as lung cancer could also be impressive.

## Author contributions

**Conceptualization:** Proloy Kumar Mondal, Haewon Byeon.

**Formal analysis:** Proloy Kumar Mondal, Md. Khurshid Jahan.

**Funding acquisition:** Haewon Byeon.

**Investigation:** Proloy Kumar Mondal, Haewon Byeon.

**Methodology:** Proloy Kumar Mondal, Md. Khurshid Jahan, Haewon Byeon.

**Project administration:** Haewon Byeon.

**Resources:** Proloy Kumar Mondal.

**Software:** Proloy Kumar Mondal, Md. Khurshid Jahan.

**Supervision:** Haewon Byeon.

**Validation:** Proloy Kumar Mondal, Haewon Byeon.

**Visualization:** Proloy Kumar Mondal, Md. Khurshid Jahan.

**Writing – original draft:** Proloy Kumar Mondal, Haewon Byeon.

**Writing – review & editing:** Proloy Kumar Mondal, Haewon Byeon.
